# Current Status of Bowel Preparation for Colonoscopy in Japan: A Web‐Based Survey of Endoscopists (PRESS Survey)

**DOI:** 10.1002/deo2.70398

**Published:** 2026-08-11

**Authors:** Takayuki Matsumoto, Motohiro Esaki, Fumihito Hirai, Minami Umeyama, Takahiro Suzuki, Toshiki Takatsuki, Yusuke Shimada, Akira Oota, Masato Ueno

**Affiliations:** ^1^ Department of Internal Medicine Division of Gastroenterology, School of Medicine Iwate Medical University Yahaba Japan; ^2^ Department of Internal Medicine Division of Gastroenterology, Faculty of Medicine Saga University Saga Japan; ^3^ Department of Gastroenterology, Faculty of Medicine Fukuoka University Fukuoka Japan; ^4^ Medical Department EA Pharma Tokyo Japan; ^5^ Pharmaceutical Development Department Data Science Group, EA Pharma Tokyo Japan

**Keywords:** cathartics, colonoscopy, constipation, laxatives, questionnaire

## Abstract

**Objectives:**

The increasing number of patients with colorectal cancer underscores the importance of colonoscopy. Adequate bowel preparation is essential to ensure the quality and safety of colonoscopic examinations; however, data on the current practices in Japan remain limited. This study aimed to clarify the current status of bowel preparation, use of bowel‐cleansing agents, and associated challenges among physicians performing colonoscopies.

**Methods:**

In March 2026, we conducted a nationwide web‐based cross‐sectional survey of physicians performing colonoscopies in Japan. The survey assessed bowel preparation practices, use of bowel‐cleansing agents, constipation, and renal function before colonoscopy. Responses were obtained from 300 physicians who performed at least 10 colonoscopies per month.

**Results:**

Low‐volume polyethylene glycol (PEG) plus ascorbate preparations were the most commonly used bowel‐cleansing agents, followed by high‐volume PEG preparations and magnesium‐based agents. Cleansing efficacy and safety were the most highly valued characteristics of bowel‐cleansing agents. 64.7% of physicians reported that the perceived frequency of inadequate bowel preparation was >10% among their patients. Although 64.3% of the physicians reported always assessing constipation, only 32.7% reported always evaluating renal function before colonoscopy. Compared with hospital‐based physicians, those working in clinics reported a significantly lower proportion of inadequate bowel preparation, but a significantly lower rate of renal function assessment.

**Conclusion:**

Although cleansing efficacy and safety are prioritized in bowel preparation for colonoscopy in Japan, physicians perceived inadequate bowel preparation to occur in a substantial proportion of patients. Thus, further optimization of bowel preparation strategies and more consistent assessment of renal function before colonoscopy are needed.

**Trial Registration:**

University Hospital Medical Information Network (UMIN000060808).

## 1 Introduction

Colorectal cancer remains a leading cause of cancer incidence and mortality worldwide [1, 2, 3]. Colonoscopy plays a crucial role in the early detection and prevention of colorectal cancer [4, 5] and is essential for assessing disease activity in conditions such as inflammatory bowel disease [6, 7]. Appropriate bowel preparation is essential to ensure diagnostic accuracy and procedural safety. In particular, bowel cleansing quality influences lesion detection rates, examination time, and the incidence of procedure‐related adverse events [8, 9, 10].

In recent years, various bowel‐cleansing agents have become available in Japan, expanding the options for bowel preparation [11]. However, the selection of preparation regimens is often based on institutional practices, physician experience, and patient characteristics, resulting in considerable variability in bowel preparation methods and evaluations in clinical practice [12, 13].

Although multiple factors are reportedly associated with inadequate bowel preparation, constipation is among the most common and clinically important patient‐related factors [14, 15, 16]. Therefore, appropriate assessment of constipation before colonoscopy and adjustment of bowel preparation strategies, when necessary, are essential to achieve adequate bowel cleansing.

Renal function should be considered when administering bowel‐cleansing agents. Certain agents have been associated with electrolyte disturbances and renal impairment, making preprocedural assessment of renal function important for ensuring procedural safety [11, 17, 18]. However, the extent to which renal function is routinely evaluated in clinical practice remains unclear.

Furthermore, Japan is experiencing rapid population aging [19], and the proportion of older patients undergoing colonoscopy continues to increase [20]. Older adults are more likely to have constipation and impaired renal function [21, 22], which may increase the risk of bowel preparation. Consequently, the optimization of bowel preparation management will become an increasingly important clinical challenge.

Against this background, a comprehensive understanding of current bowel preparation practices in Japan, particularly regarding the use of bowel‐cleansing agents and the assessment of constipation and renal function, is clinically meaningful. Therefore, we conducted a web‐based questionnaire survey of physicians performing gastrointestinal endoscopy, designated the Preparation for Endoscopic Screening Survey (PRESS survey), to clarify the current status of bowel preparation for colonoscopy and identify the remaining challenges in clinical practice.

## 2 Methods

### 2.1 Survey Instrument

This study was conducted using a web‐based, cross‐sectional questionnaire survey targeting physicians who perform colonoscopies in Japan. The survey was commissioned by EA Pharma Co., Ltd. (Tokyo, Japan) and administered by Cross Marketing, Inc. (Tokyo, Japan). The questionnaire was developed by the study investigators prior to survey implementation. The study protocol was registered with the University Hospital Medical Information Network (UMIN000060808).

On March 13, 2026, invitations were distributed to 18,672 registered physicians nationwide through an online research panel, irrespective of specialty. Physicians who reported performing at least 10 colonoscopies per month were considered eligible for participation. Among respondents who met these criteria, data were consecutively collected until a predefined sample size of 300 physicians was reached. No respondents were excluded from the analysis.

The survey collected information on physician and practice characteristics, including age, sex, type of medical institution, academic society affiliation, and the number of endoscopic procedures performed. Physicians working in screening centers were included in the clinic‐based group for facility‐based comparisons. In addition, detailed information regarding bowel preparation practices was obtained, including the use of sedative agents, prescription of additional laxatives on the day before colonoscopy, use of preprocedure diets, location of bowel cleansing agent administration, types of bowel cleansing agents used, factors prioritized when selecting bowel cleansing agents, and the perceived proportion of inadequate bowel preparation.

The survey also assessed constipation and renal function before colonoscopy, including their perceived impact on bowel preparation. Constipation and impaired renal function were assessed according to physicians’ routine clinical judgment without predefined study‐specific criteria. Information regarding the assessment of renal function, the proportion of patients with impaired renal function, and the proportions of patients aged ≥65 years and ≥75 years undergoing colonoscopy was also collected. The full questionnaire is provided in Appendix S1.

### 2.2 Statistical Analysis

All statistical analyses were performed using the SAS (version 9.4; SAS Institute Japan Ltd.). Categorical variables were compared using Fisher's exact test, including comparisons between hospital‐based and clinic‐based physicians. Mean importance scores for each characteristic of bowel‐cleansing agents, assessed on an 11‐point scale by the same respondents, were compared using the paired t‐test. All statistical tests were two‐sided, and a *p*‐value of < 0.05 was considered statistically significant.

### 2.3 Ethical Considerations

This survey was approved by the Ethics Committee of the Yamauchi Clinic (approval no. 2026‐02‐00388). This study was conducted in accordance with the principles of the Declaration of Helsinki. Informed consent for participation in the study was obtained electronically from all participants via the web‐based survey system.

## 3 Results

### 3.1 Physician Characteristics

Responses were obtained from 300 physicians who consented to participate. No responses were considered inappropriate; therefore, all the responses were included in the analysis. The mean age of the respondents was 50.0 years (median, 50 years). The majority of respondents were male (93.7%). Gastroenterologists, gastrointestinal surgeons, and general internists accounted for 76.3%, 18.0%, and 5.7% of the cases, respectively. The membership rates of the Japanese Society of Gastroenterology and the Japan Gastroenterological Endoscopy Society were 91.7% and 93.3%, respectively (Table 1). The mean number of colonoscopies performed per month was 40.5 (median, 30).

**TABLE 1:** Physician characteristics.

Characteristics	*N* = 300
Age, years	50.0 ± 10.9 (median, 50)
Male, *n* (%)	281 (93.7)
Specialty	
Gastroenterology	229 (76.3)
Gastrointestinal surgery	54 (18.0)
General internal medicine	17 (5.7)
Proctology	0 (0.0)
Other	0 (0.0)
Affiliation (facility type)	
University hospital	45 (15.0)
General hospital	180 (60.0)
Clinic	65 (21.7)
Screening center	10 (3.3)
None	0 (0.0)
Membership in professional societies	
Japanese Society of Gastroenterology	275 (91.7)
Japan Gastroenterological Endoscopy Society	280 (93.3)
Number of colonoscopies performed per month	40.5 ± 39.2 (median, 30)

Data are presented as means ± standard deviations (medians) or *n* (%).

### 3.2 Practices Related to Colonoscopy Preparation

**FIGURE 1:**
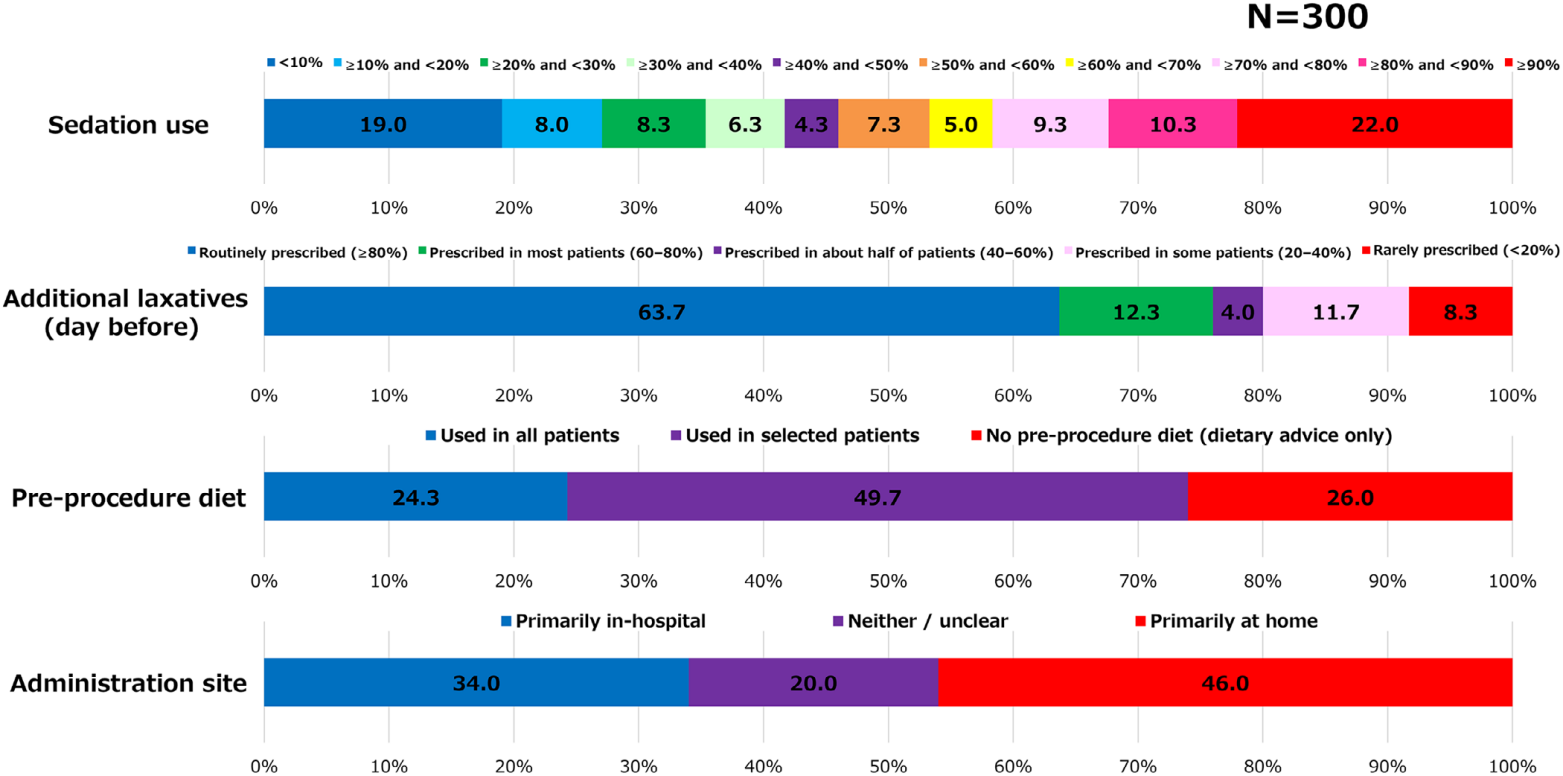
Practices related to colonoscopy preparation. The proportion of patients undergoing sedation, the proportion of patients prescribed additional laxatives on the day before colonoscopy, use of pre‐procedure diets on the day before colonoscopy, and location of bowel‐cleansing agent administration (primarily in‐hospital or at home) are shown.

Practices related to bowel preparation for colonoscopy are shown in Figure 1. The proportion of patients who undergo sedation varies widely among physicians. Nineteen percent of physicians reported using sedation in <10% of patients, whereas 22.0% reported routinely using sedation in >90% of patients. Regarding the prescription of additional laxatives on the day before colonoscopy, 63.2% of physicians reported that they routinely prescribed them for most patients (≥80%). Regarding the use of pre‐procedure diets the day before colonoscopy, 24.3% of the physicians reported using them for all patients, whereas 26.0% reported not using pre‐procedure diets. Regarding the location of bowel‐cleansing agent administration, 46.0% of physicians reported that bowel‐cleansing agents were generally taken at home, whereas 34.0% reported that administration was generally performed in the hospital.

### 3.3 Use of Bowel‐cleansing Agents

**FIGURE 2:**
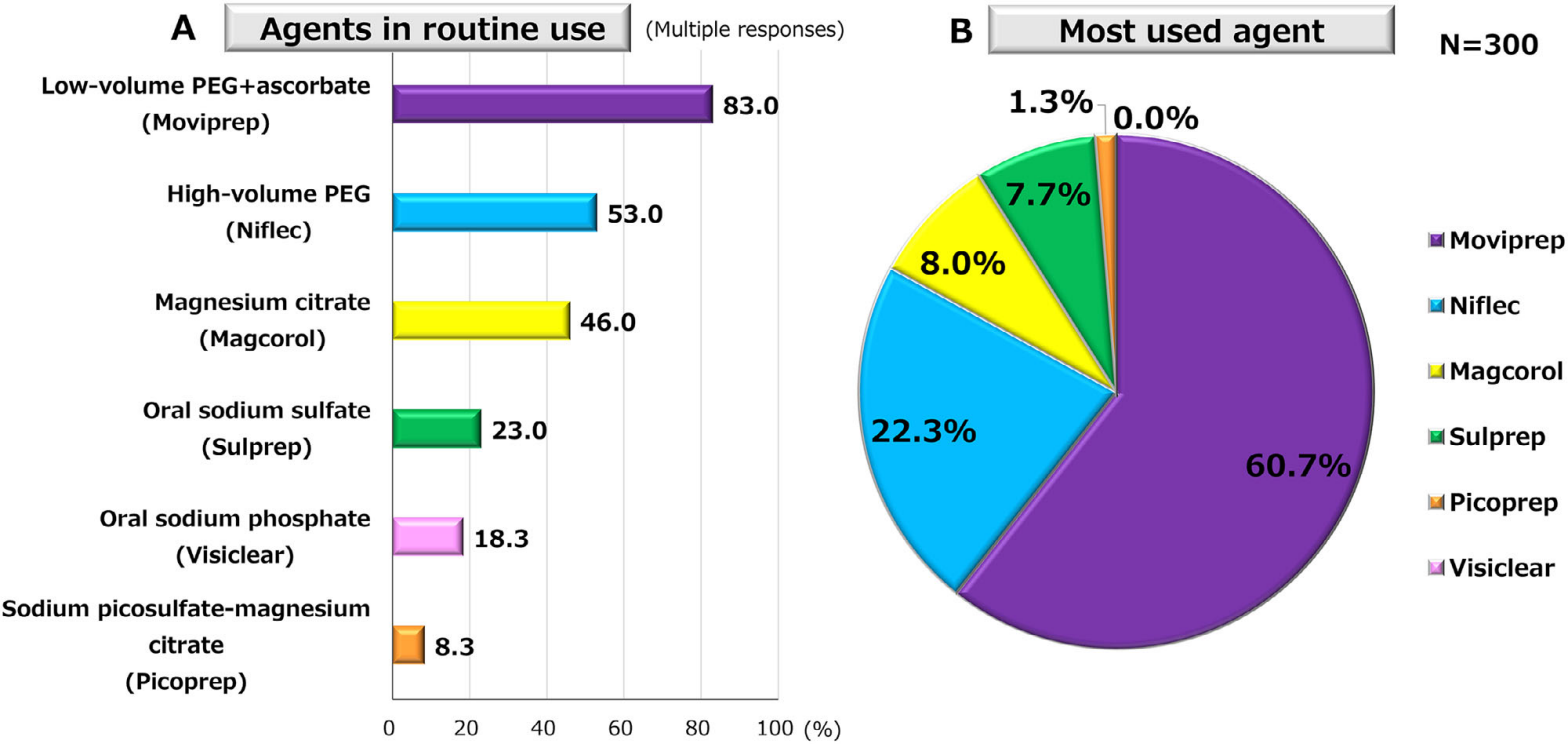
Use of bowel cleansing agents. The use of bowel cleansing agents is shown as follows: (A) Bowel‐cleansing agents used in routine clinical practice (multiple responses were allowed). (B) The bowel‐cleansing agent was used most frequently (single‐choice response).

The use of bowel‐cleansing agents is illustrated in Figure 2. Low‐volume polyethylene glycol (PEG) plus ascorbate preparations were the most commonly used bowel‐cleansing agents in routine clinical practice (83.0%), followed by high‐volume PEG preparations (53.0%) and magnesium citrate preparations (46.0%). When asked regarding the bowel‐cleansing agent used most frequently, 60.7% of physicians reported low‐volume PEG plus ascorbate preparations, followed by high‐volume PEG preparations (22.3%) and magnesium‐based preparations (8.0%).

### 3.4 Desired Characteristics of Bowel‐cleansing Agents

**FIGURE 3:**
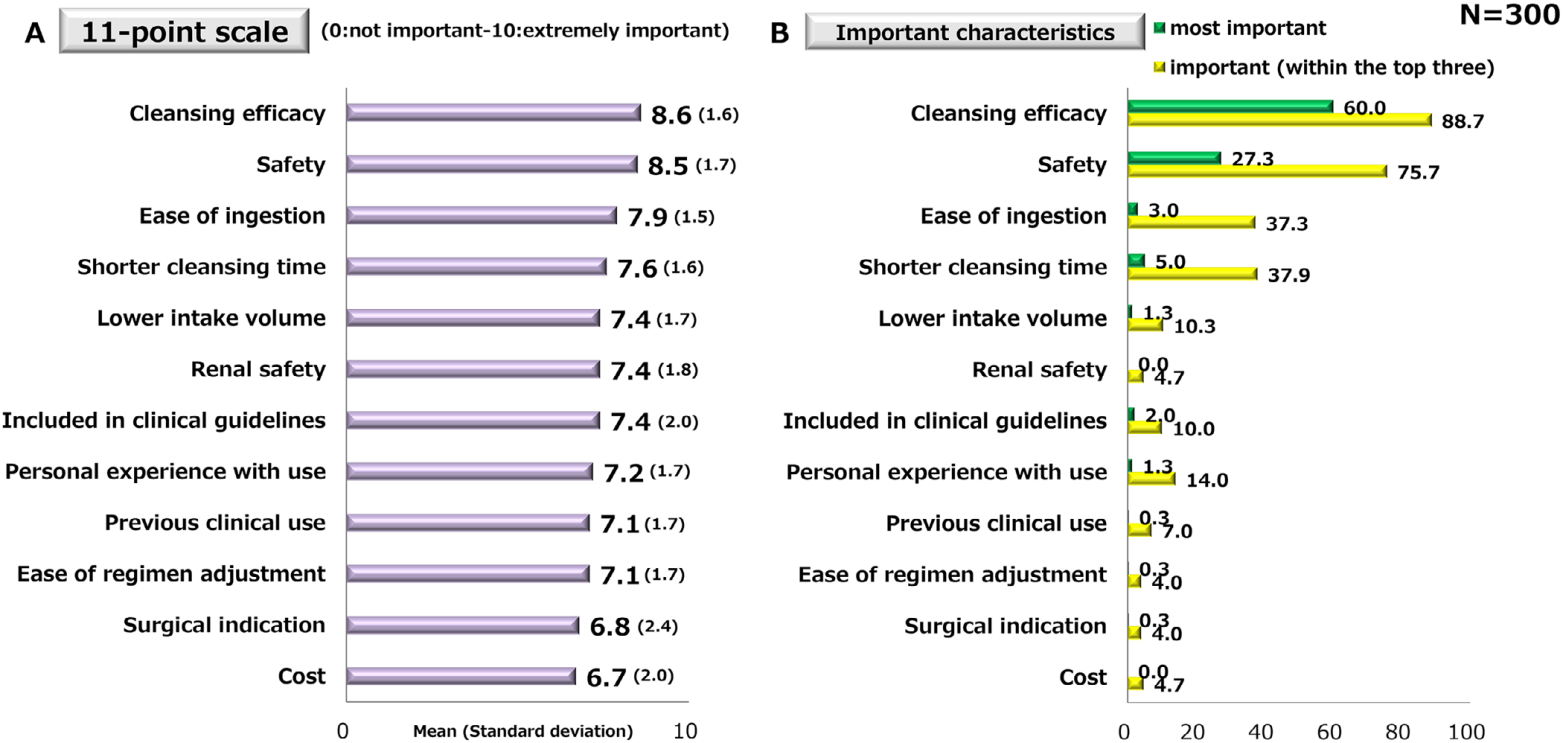
Desired characteristics of bowel cleansing agents. Characteristics considered important by physicians when selecting bowel cleansing agents are shown as follows: (A) The mean importance scores for each characteristic were assessed using an 11‐point scale (0 = not important to 10 = extremely important). (B) The proportion of physicians who selected each characteristic as “most important” or “important (within the top three).” On the 11‐point rating scale, the mean scores for cleansing efficacy and safety were significantly higher than those for all the other characteristics (*p* < 0.0001). The third‐highest rated characteristic, ease of ingestion, was also significantly higher than all the other characteristics (*p* < 0.0001). Statistical significance was assessed using a paired t‐test.

The characteristics considered important when selecting bowel‐cleansing agents are shown in Figure 3. On the 11‐point rating scale, the mean scores for cleansing efficacy (8.6) and safety (8.5) were the highest and were significantly greater than those for all other evaluated characteristics (*p* < 0.0001). The third‐highest rated characteristic, ease of ingestion (7.9), was also rated significantly higher than all the other characteristics (*p* < 0.0001). When considering the proportion of physicians who selected each characteristic as most important, cleansing efficacy (60.0%) and safety (27.3%) were selected most frequently.

### 3.5 Perceived Proportion of Patients With Inadequate Bowel Preparation

**FIGURE 4:**
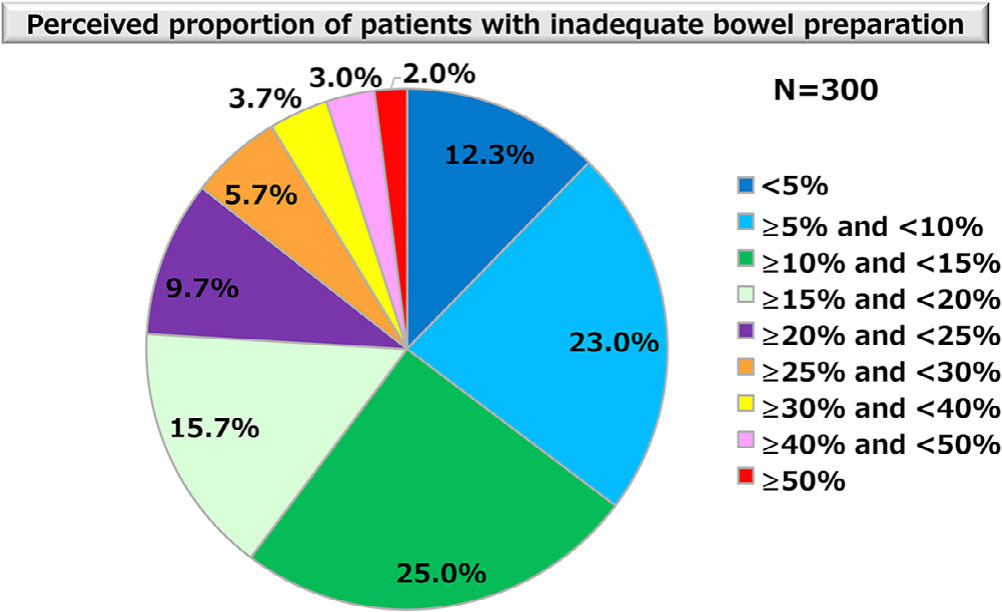
Perceived proportion of patients with inadequate bowel preparation. The distribution of responses regarding the perceived proportion of patients with inadequate bowel preparation in routine clinical practice is shown.

The perceived proportion of patients with inadequate bowel preparation is shown in Figure 4. Although 35.3% of physicians reported that the perceived frequency of inadequate bowel preparation was <10% among their patients, 64.7% reported that the perceived frequency was ≥10%. Approximately one‐quarter of physicians reported that the perceived frequency of inadequate bowel preparation was ≥20%.

### 3.6 Assessment and Impact of Constipation Before Colonoscopy

**FIGURE 5:**
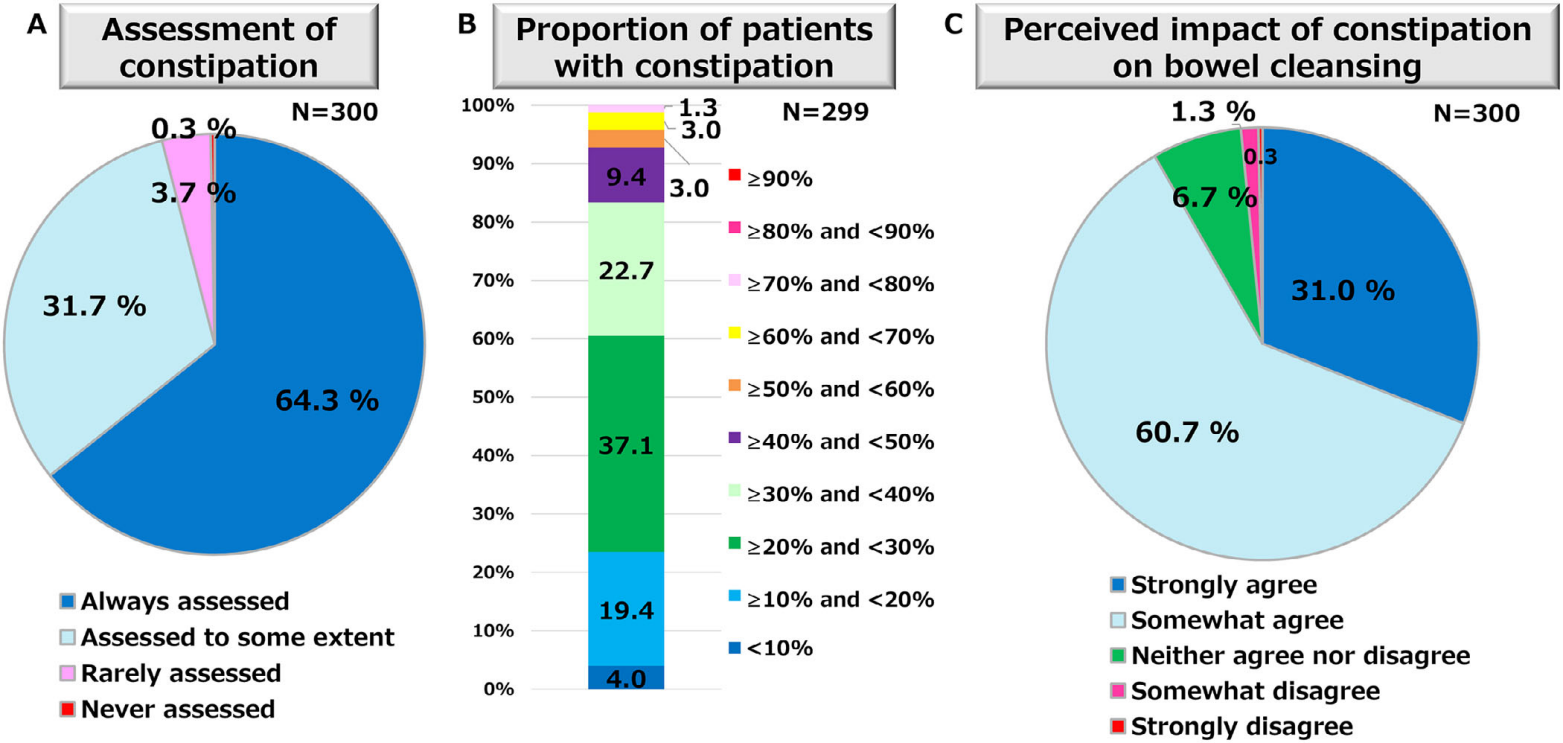
Assessment of constipation before colonoscopy and its impact. (A) Assessment of constipation before colonoscopy. (B) Distribution of responses regarding the proportion of patients with constipation (excluding one physician who reported not assessing constipation at all). (C) Physicians’ perceptions of whether patients with constipation are more likely to have inadequate bowel preparation.

The assessment of constipation before colonoscopy and its perceived impact are shown in Figure 5. Overall, 64.3% of physicians reported that they always assessed the presence of constipation before colonoscopy, and this proportion increased to 96.0% when physicians who reported assessing constipation to some extent were included. Regarding the impact of constipation on bowel preparation quality, 91.7% of the physicians reported that they strongly agreed or partly agreed that patients with constipation were more likely to have inadequate bowel preparation. Regarding the proportion of patients with constipation, 69.5% of physicians reported that ≥20% of their patients who underwent colonoscopy had constipation.

### 3.7 Assessment of Renal Function and Proportion of Older Patients

**FIGURE 6:**
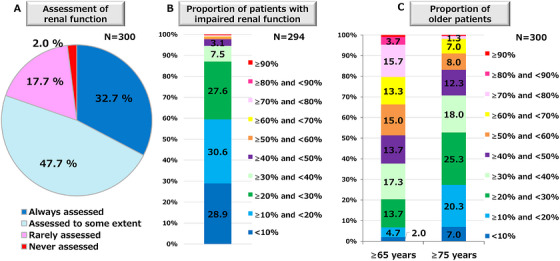
Assessment of renal function and proportion of older patients. (A) Assessment of renal function before colonoscopy. (B) Distribution of responses regarding the proportion of patients with impaired renal function (excluding six physicians who reported not assessing renal function at all). (C) Distribution of responses regarding the proportion of older patients (aged ≥65 years and ≥75 years).

The assessment of renal function before colonoscopy and the proportion of older patients are shown in Figure 6. Overall, 32.7% of the physicians reported that they always assessed renal function before colonoscopy, whereas 19.7% reported that they rarely or never assessed renal function. Regarding patient age distribution, 48.6% of physicians reported that patients aged ≥65 years accounted for >50% of the individuals undergoing colonoscopy. In addition, 47.4% reported that patients aged ≥75 years accounted for ≥30% of their colonoscopy population.

### 3.8 Comparison Between Hospital‐based and Clinic‐based Physicians

**FIGURE 7:**
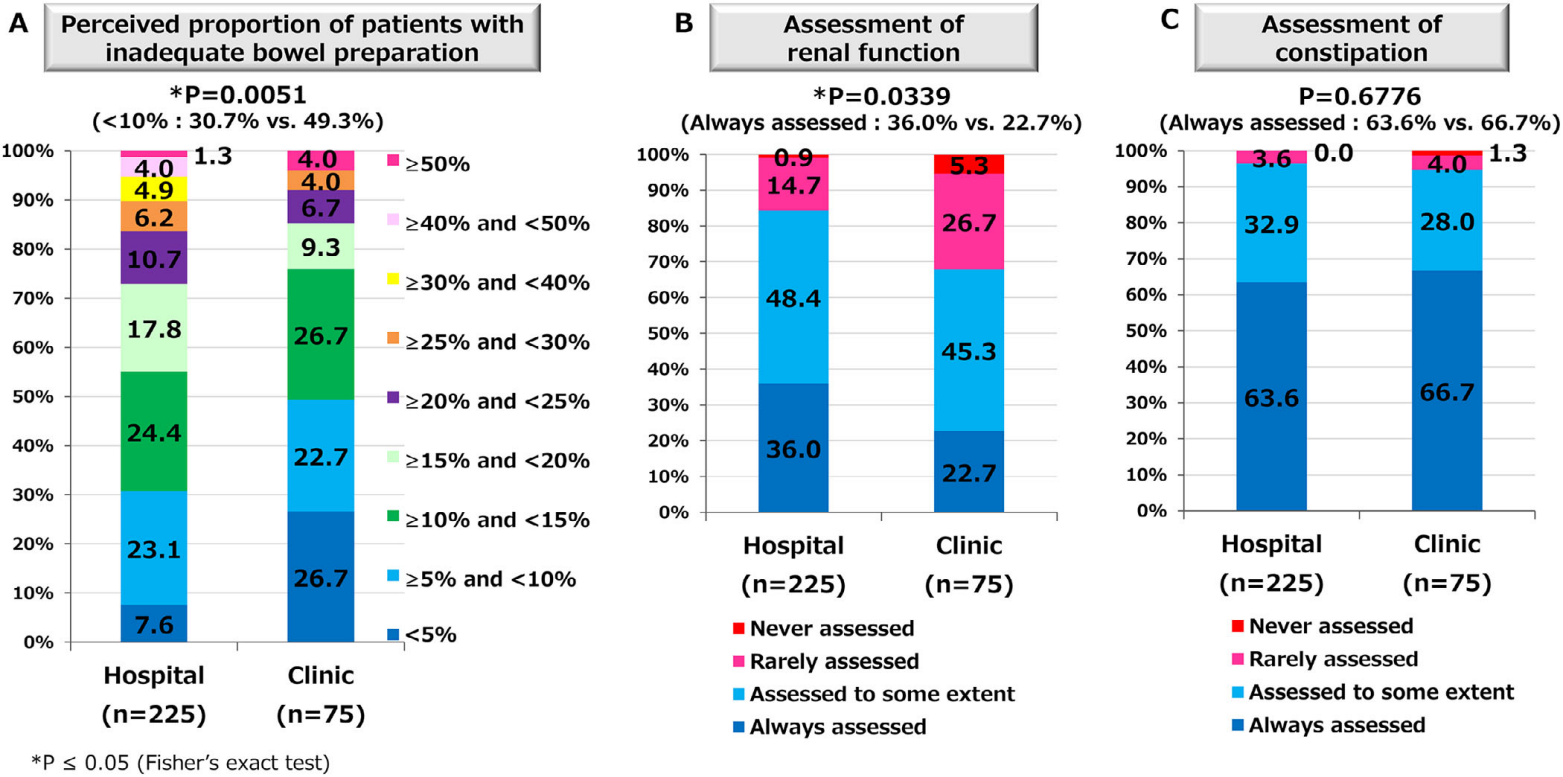
Comparison between hospital‐based and clinic‐based physicians. Comparisons between hospital‐based physicians (*n* = 225) and clinic‐based physicians (*n* = 75), including physicians working in screening centers, were as follows: (A) Distribution of the perceived proportion of patients with inadequate bowel preparation. (B) Renal function assessment. (C) Constipation assessment. Between‐group comparisons were performed using Fisher's exact test.

The results of the comparisons between hospital‐based physicians (*n* = 225) and clinic‐based physicians, including those working in screening centers (*n* = 75), are shown in Figure 7. The proportion of physicians who reported a perceived frequency of inadequate bowel preparation of <10% was significantly higher among clinic‐based physicians than among hospital‐based physicians (49.3% vs. 30.7%, *p* = 0.0051). By contrast, the proportion of physicians who reported that they always assessed renal function before colonoscopy was significantly higher among hospital‐based physicians than among clinic‐based physicians (36.0% vs. 22.7%, *p* = 0.0339). No significant difference was observed in the assessment of constipation before colonoscopy between the two groups.

## 4 Discussion

This web‐based survey of 300 physicians (PRESS survey) provides an overview of bowel preparation practices, bowel cleansing agent use, and key factors including constipation, renal function, and older patients. The major findings of this study were as follows: (1) cleansing efficacy and safety were the most highly prioritized characteristics when selecting bowel‐cleansing agents; (2) more than half of physicians perceived inadequate bowel preparation in ≥10% of patients; (3) constipation assessment was widely performed, whereas routine renal function assessment was less common.

Sedation practices varied widely, with some routinely using sedation and others rarely. Its use requires careful consideration of patient characteristics and resources [23]. Consistent with reports from Western countries, our findings confirmed the marked interfacility variation in sedation practices in Japan [24]. Regarding the prescription of additional laxatives on the day before colonoscopy, most physicians (63.2%) routinely prescribed them to most patients. Although the effectiveness of adding laxatives the day before colonoscopy has been investigated in several studies, no clear consensus on the optimal choice of agent has been established [25, 26, 27, 28, 29]. As our survey did not assess the specific types or effectiveness of these laxatives, further research is needed to clarify the role and optimal selection of additional laxatives.

The usefulness of pre‐procedural diets has been reported [30, 31]. Although guidelines recommend a low‐residue diet, no clear standards exist for commercially prepared diets [10], leaving their use to physician discretion. This likely explains the wide variability in pre‐procedural diet use observed in our survey. Regarding the location of bowel cleansing agent administration, home‐based administration was slightly more common; however, a previous study has reported shorter bowel cleansing times with in‐hospital administration, which should be considered [32].

In Japan, several bowel‐cleansing agents have become available over time, including magnesium citrate (Magcorol) in 1988, high‐volume PEG (Niflec) in 1992, oral sodium phosphate (Visiclear) in 2007, low‐volume PEG plus ascorbate (Moviprep) in 2013, sodium picosulfate–magnesium citrate (Picoprep) in 2016, and oral sodium sulfate (Sulprep) in 2023 [11]. In this study, low‐volume PEG plus ascorbate preparations were most commonly used, followed by high‐volume PEG, magnesium citrate, and oral sodium sulfate. The most highly valued characteristics of bowel‐cleansing agents were cleansing efficacy and safety, both of which were rated significantly higher than other characteristics. These were most frequently selected, indicating that effectiveness and safety are the highest priorities in practice in Japan. This finding is consistent with international guideline recommendations that emphasize the prioritization of efficacy over tolerability given that inadequate bowel preparation can lead to missed lesions and the need for repeat examinations [10, 33]. Ease of ingestion was ranked as the third most important characteristic and rated significantly higher than the other characteristics. Thus, while efficacy and safety are fundamental, patient tolerability and completion are also important. By contrast, patient‐centered studies conducted overseas have reported that low volume and ease of ingestion are the most highly valued factors [34], suggesting that the observed differences may reflect differing perspectives between physicians and patients. The high utilization rate of low‐volume PEG plus ascorbate preparations observed in this study is consistent with these priorities, as these agents maintain cleansing efficacy and safety comparable to high‐volume PEG, while improving tolerability through reduced intake volume [35, 36].

Regarding the perceived frequency of inadequate bowel preparation, only approximately one‐third of the physicians reported experiencing inadequate bowel preparation in <10% of patients, whereas approximately two‐thirds of physicians reported a perceived frequency of inadequate bowel preparation of ≥10%. Inadequate bowel preparation is important because it reduces lesion detection rates, prolongs examination time, and increases the likelihood of repeat procedures or additional interventions [10]. Thus, inadequate bowel preparation remains a challenge. These results highlight the need for further individualization of bowel preparation regimens based on patient characteristics and for strengthening pre‐procedural assessment and patient education. However, these findings should be interpreted with caution, as they are based on physicians’ perceptions rather than objective measurements.

The occurrence of inadequate bowel preparation is influenced not only by the type of cleansing agent used but also by patient‐related factors such as older age, constipation, diabetes mellitus, obesity, neurological disorders, and history of abdominal surgery [14, 15, 16]. Among these, constipation is a common gastrointestinal condition with a high prevalence [37, 38, 39], underscoring the importance of appropriate screening. In our survey, constipation was widely assessed, and a strong consensus among physicians was that constipation increases the risk of inadequate bowel preparation. Therefore, constipation is well recognized as a major risk factor, and its assessment has been incorporated into bowel preparation practices to some extent.

By contrast, only approximately one‐third of the physicians always reported assessing renal function before colonoscopy, and a substantial proportion reported rarely or never doing so. The assessment of renal function is an important component of safe bowel preparation, particularly when considering patient comorbidities, advanced age, concomitant medications, risks of dehydration, and electrolyte disturbances [10, 17, 40]. Given that previous reports have indicated a high prevalence of impaired renal function in aging populations [23, 41], our findings suggest that a more consistent assessment of renal function is warranted.

A considerable proportion of physicians reported that older patients aged ≥65 or ≥75 years accounted for a large share of their colonoscopy population, highlighting the growing role of older adults in endoscopic practice in Japan. As the aging population continues to grow [42], the importance of appropriate management strategies for elderly patients undergoing colonoscopy will further increase. Older patients often have factors that compromise bowel preparation, including constipation, renal impairment, dehydration, electrolyte imbalance, and frailty [14, 33, 43]. Accordingly, ensuring both safety and effectiveness of bowel preparation is critical for this population. Our finding that constipation assessment is widely performed, whereas renal function assessment shows substantial variability, underscores the need for a more systematic preprocedural evaluation and individualized bowel preparation strategies for high‐risk groups, including older adults.

In facility‐based comparisons, clinic‐based physicians more frequently reported a perceived frequency of inadequate bowel preparation of <10%, whereas hospital‐based physicians were more likely to report assessing renal function. These findings should be interpreted cautiously because no adjustment was made for potential confounding factors. The observed differences may reflect variations in patient populations and clinical settings [45, 46]. Achieving consistently high standards for bowel preparation quality and safety assessment may require standardized practices tailored to facility characteristics, improved information sharing with referring physicians, structured preprocedure evaluations, and standardized patient education [10].

This study has several limitations. The findings were based on physician‐reported assessments and may be subject to recall bias and interobserver variability. Inadequate bowel preparation was not evaluated using validated scoring systems such as the Boston Bowel Preparation Scale [47, 48]. In addition, constipation and impaired renal function were assessed according to physicians’ routine clinical judgment. Because participants were recruited through a web‐based panel and limited to physicians performing at least 10 colonoscopies per month, generalizability may be limited. Future studies using objective clinical data are warranted.

In conclusion, the PRESS survey demonstrated that cleansing efficacy and safety were the highest priorities in bowel preparation for colonoscopy in Japan, with low‐volume PEG plus ascorbate preparations being the most commonly used. However, inadequate bowel preparation is still perceived to occur at a substantial frequency. Although constipation assessment is widely implemented, gaps remain in the assessment of renal function, with variability across practice settings. In an aging population, further efforts to optimize evaluation, individualize strategies, and standardize practices are essential.

## Author Contributions

**Takayuki Matsumoto**, **Motohiro Esaki**, and **Fumihito Hirai** supervised the study and prepared the manuscript. **Minami Umeyama**, **Takahiro Suzuki**, **Toshiki Takatsuki**, and **Masato Ueno** designed the study and drafted and revised the manuscript. **Yusuke Shimada** performed the data analysis. **Akira Oota** conducted a quality check of the data analysis. All the authors have read and approved the final version of this manuscript.

## Funding

This study was supported by EA Pharma (Tokyo, Japan).

## Ethics Statement

**Approval of the research protocol**: This study was approved by the Ethics Committee of the Yamauchi Clinic (approval no. 2026‐02‐00388).

## Consent

Informed consent was obtained electronically from all participants.

## Conflicts of Interest

Takayuki Matsumoto, Motohiro Esaki, and Fumihito Hirai received lectures and consultation fees from EA Pharma. Minami Umeyama, Takahiro Suzuki, Toshiki Takatsuki, Yusuke Shimada, Akira Oota, and Masato Ueno are current employees of EA Pharma. EA Pharma was involved in the study design, questionnaire development, data analysis, and interpretation of the results. The survey was conducted by Cross Marketing Inc. under commission from EA Pharma. The authors had full and independent access to the data and take responsibility for the integrity of the data and accuracy of the analysis. The academic authors critically reviewed the data interpretation and contributed to the manuscript preparation. The decision to submit the manuscript for publication was made solely by the authors.

## Supporting information




**APPENDIX S1** Questionnaire used in the PRESS survey.
The full questionnaire, including all items and response options administered in Japanese and translated into English, is provided.

## Data Availability

These data are not available publicly. The data used and analyzed in this study are available from the corresponding author upon reasonable request.
