# Supplementary material for: Current Status of Bowel Preparation for Colonoscopy in Japan: A Web‐Based Survey of Endoscopists (PRESS Survey)
Source: DEN Open. 2026 Aug 11;7(1):e70398. doi: 10.1002/deo2.70398 (PMC13458466; doi:10.1002/deo2.70398)
Supplement: Supplementary file 1 [file DEO2-7-e70398-s001.docx]

Supplementary Appendix 1. Questionnaire used in the PRESS survey

# 1. Physician characteristics

1. Age (years)

- Enter numerical value

2. Sex

- Male
- Female

3. Specialty

- Gastroenterology
- Gastrointestinal surgery
- General internal medicine
- Proctology
- Other

4. Type of institution

- University hospital
- General hospital
- Clinic
- Screening center

5. Membership in professional societies (multiple answers allowed)

- Japanese Society of Gastroenterology
- Japan Gastroenterological Endoscopy Society
- Other

6. Number of colonoscopies performed per month

- Enter numerical value

# 2. Colonoscopy preparation practices

7. Proportion of patients receiving sedation

- <10%
- ≥10% and <20%
- ≥20% and <30%
- ≥30% and <40%
- ≥40% and <50%
- ≥50% and <60%
- ≥60% and <70%
- ≥70% and <80%
- ≥80% and <90%
- ≥90%

8. Frequency of additional laxatives use (day before)

- ≥80%
- 60–80%
- 40–60%
- 20–40%
- <20%

9. Use of pre-procedure diet

- Used in all patients
- Used in selected patients
- No pre-procedure diet

10. Administration site of bowel preparation

- Primarily in hospital
- Primarily at home
- Neither / unclear

# 3. Use of bowel-cleansing agents

11. Agents used (multiple answers allowed)

- Low-volume PEG + ascorbate
- High-volume PEG
- Magnesium citrate
- Oral sodium sulfate
- Sodium picosulfate–magnesium citrate
- Oral sodium phosphate

12. Most frequently used agent (single answer)

- Low-volume PEG + ascorbate
- High-volume PEG
- Magnesium citrate
- Oral sodium sulfate
- Sodium picosulfate–magnesium citrate
- Oral sodium phosphate

# 4. Factors influencing choice

13. Importance of characteristics (0=not important – 10=extremely important)

- Cleansing efficacy
- Safety
- Ease of ingestion
- Shorter cleansing time
- Lower intake volume
- Renal safety
- Guideline inclusion
- Personal experience
- Previous clinical use
- Cost

14. Select top three characteristics

- Cleansing efficacy
- Safety
- Ease of ingestion
- Shorter cleansing time
- Lower intake volume
- Renal safety
- Guideline inclusion
- Personal experience

# 5. Perceived frequency of inadequate bowel preparation

15. Perceived proportion of patients with inadequate bowel preparation

- <5%
- ≥5% and <10%
- ≥10% and <15%
- ≥15% and <20%
- ≥20% and <25%
- ≥25% and <30%
- ≥30% and <40%
- ≥40% and <50%
- ≥50%

# 6. Assessment of constipation

16. Constipation assessment frequency

- Always
- To some extent
- Rarely
- Never

17. Proportion of patients with constipation

- <10%
- 10–20%
- 20–30%
- 30–40%
- 40–50%
- ≥50%

18. Impact of constipation on bowel preparation

- Strongly agree
- Somewhat agree
- Neutral
- Somewhat disagree
- Strongly disagree

# 7. Renal function and patient characteristics

19. Renal function assessment frequency

- Always
- To some extent
- Rarely
- Never

20. Proportion of patients with impaired renal function

- <10%
- 10–20%
- 20–30%
- 30–40%
- ≥40%

21. Proportion of patients aged ≥65 years

- <10%
- 10–20%
- 20–30%
- 30–40%
- 40–50%
- 50–60%
- 60–70%
- 70–80%
- 80–90%
- ≥90%

22. Proportion of patients aged ≥75 years

- <10%
- 10–20%
- 20–30%
- 30–40%
- 40–50%
- 50–60%
- 60–70%
- 70–80%
- 80–90%
- ≥90%
